# Hierarchically Restructured Antibacterial Electrodes for Neural Interfaces: Electrochemical and Microstructural Evolution under Extended Cycling

**DOI:** 10.1021/acsami.5c21727

**Published:** 2026-03-16

**Authors:** Kriti Panchal, Wesley Seche, Henna Khosla, Gang Feng, Jacob Elmer, Gregory A. Caputo, Steven J. May, Ekaterina Pomerantseva, Shahram Amini

**Affiliations:** † Department of Materials Science and Engineering, 6527 Drexel University, Philadelphia, Pennsylvania 19104, United States; ‡ Pulse Technologies Inc. (An Integer Holdings Company), Quakertown, Pennsylvania 18951, United States; § Department of Mechanical Engineering, 8210 Villanova University, Villanova, Pennsylvania 19085, United States; ∥ Department of Chemical and Biological Engineering, Villanova University, Villanova, Pennsylvania 19085, United States; ⊥ Biomedical Engineering Department, University of Connecticut, Storrs, Connecticut 06269, United States; # Department of Chemistry and Biochemistry, 3536 Rowan University, Glassboro, New Jersey 08028, United States

**Keywords:** hierarchical surface restructuring, antibacterial coatings, neural interfacing, electrodes, platinum–iridium, zinc oxide films, electrochemically active surface area

## Abstract

Hierarchically restructured platinum–iridium electrodes offer high electrochemical performance for neurostimulation and cardiac rhythm management devices but require added antibacterial functionality to reduce postsurgical infection risks. In this work, electrochemically active antibacterial platinum–iridium electrodes were developed using a two-step process. First, the electrodes were restructured using a femtosecond laser hierarchical surface restructuring. In the second step, reactive magnetron sputtering from a pure zinc target in an Ar/O_2_ gas mixture was employed to deposit antibacterial zinc oxide (ZnO) thin films onto the hierarchical surface structure of the electrodes, thereby imparting antibacterial properties. X-ray diffraction and X-ray photoelectron spectroscopy confirmed the formation of ZnO. The electrochemical performance of the electrodes increased with the ZnO film deposition time. This enhancement is attributed to the nonconformal nature of the ZnO layer over the complex electrode topography, as revealed by scanning electron microscopy (SEM). SEM imaging combined with energy-dispersive spectroscopy (EDS) mapping after electrochemical cycling revealed the gradual dissolution of ZnO into the electrolyte and the recrystallization of ZnO on the electrode surface after 1,500 cyclic voltammetry (CV) cycles (24 h), likely due to the confined electrolyte environment. Electrodes coated with ZnO films exhibited significant antibacterial activity against *Escherichia coli* and *Staphylococcus aureus* bacterial strains *in vitro*. The findings of this work highlight a promising strategy for developing multifunctional, electrochemically active antibacterial electrodes for next-generation neural interfacing electrodes.

## Graphical abstract

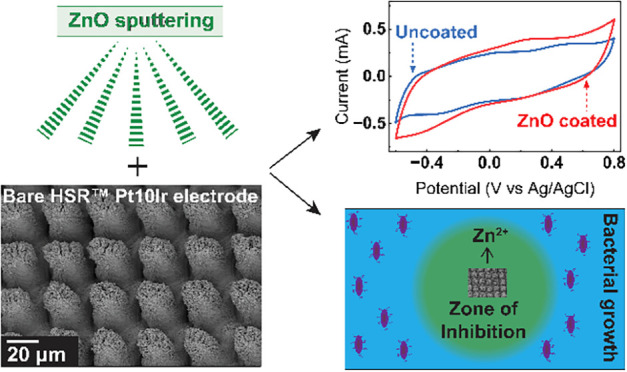

## 1 Introduction

Long-term implantable medical devices represent a rapidly advancing frontier in modern medicine, with their development requiring precise optimization of material properties and functional parameters to ensure long-term efficacy and reliability. Among these technologies, neurostimulation^1–4^ and cardiac rhythm management (CRM)^5,6^ devices play a critical role in the treatment of a wide range of neurological and cardiac disorders by delivering targeted electrical stimulation to specific sites within the central or peripheral nervous system or the myocardium, thereby modulating biological activity through inhibition, excitation, or alteration of native signal pathways.

This stimulation is achieved by transferring externally generated electrical signals from a neurostimulator or an implantable pulse generator (IPG) via a lead to an implantable electrode or microelectrode array, thereby inducing controlled changes in neural activity.^7^ Among these system components, electrodes and microelectrode arrays serve as the primary interface between the device and the biological environment, playing a pivotal role in ensuring the precise, efficient, and safe delivery of electrical stimuli. A critical requirement for electrodes used in neural interfaces such as neurostimulation and CRM applications is their electrochemical performance, which governs both the fidelity of neural modulation (or cardiac pacing) and the long-term stability of the device.

Hierarchically restructured platinum–iridium (Pt10Ir) electrodes, fabricated using femtosecond-laser hierarchical surface restructuring (HSR) technology, have recently demonstrated exceptional electrochemical performance in previous studies,^8–10^ making them highly promising for advanced neural interfacing applications. Their enhanced electrochemical activity is attributed to the engineered hierarchical surface topography, consisting of primary micropillar structures approximately 10–20 μm in height, overlaid with secondary nanoscale features ranging from a few nanometers to several hundred nanometers. This multiscale architecture significantly increases the electrochemical surface area (ESA) while preserving geometric compactness, thereby improving both the charge transfer efficiency and signal fidelity. This hierarchical architecture significantly enhances the ESA without increasing the overall electrode footprint, resulting in marked improvements in both specific capacitance and charge storage capacity.^8,10^ However, despite their superior electrochemical characteristics, HSR-processed electrodes inherently lack antibacterial properties, necessitating additional surface modifications to impart antimicrobial functionality without compromising their electrochemical performance.

Antibacterial functionality remains a critical design consideration across all implantable medical devices. Following implantation, these devices are often recognized as foreign bodies by the immune system, triggering inflammatory responses that can facilitate bacterial and biofilm colonization.^11^ Despite the use of immunosuppressive strategies, postimplantation infections occur in approximately 20% of patients,^12–16^ often resulting in serious complications and postsurgical hospitalizations. Figure 1 illustrates representative examples of implantable devices commonly used in clinical practice, along with a schematic illustration of the biofilm formation mechanism that can occur following implantation.

**Figure 1:**
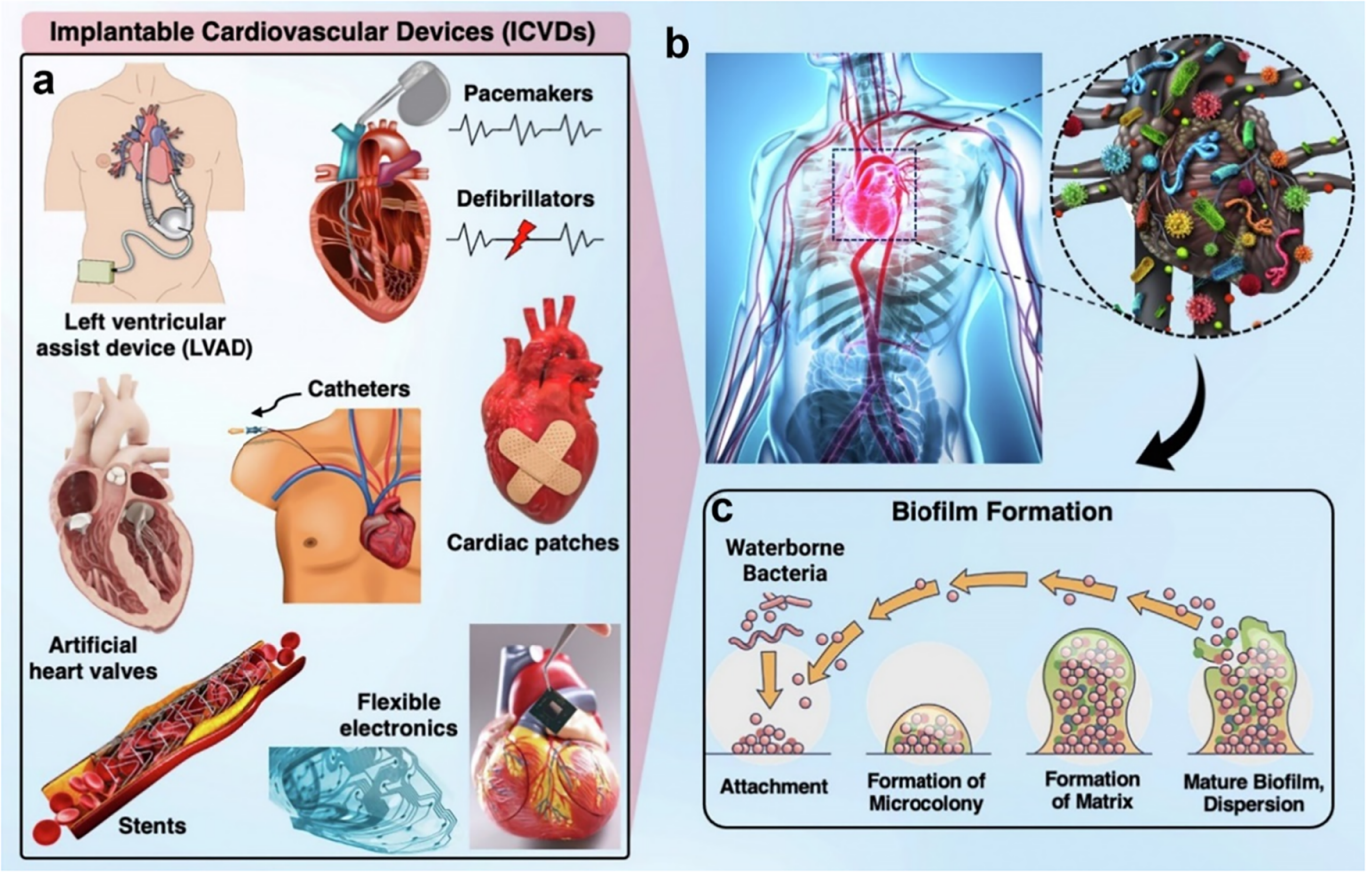
(a) and (b) Examples of implantable cardiovascular devices prone to biofilm-related infections, including stents, pacemakers, defibrillators, heart valves, cardiac patches, and flexible electronics. (c) Schematic of biofilm formation mechanism. Reprinted from Current Opinion in Biomedical Engineering,^17^ Vol. 23, E. Mostafavi, A.K. Dubey, B. Walkowiak, A. Kaushik, S. Ramakrishna, L. Teodori, “Antimicrobial surfaces for implantable cardiovascular devices,” p. 100406, © 2022 Elsevier, with permission.

## 2 Objectives

This study focuses on addressing the critical need to balance electrochemical performance and antibacterial activity in implantable electrodes for neural interfacing applications. One promising strategy for achieving this balance is the incorporation of antibacterial surface coatings.^18,19^ Among the materials investigated, zinc (Zn), silver (Ag), and copper (Cu)-based coatings have been extensively studied for their broad-spectrum antibacterial efficacy against various bacterial strains.^17,20–22^ Of these, zinc oxide (ZnO) has emerged as a particularly attractive candidate for implant applications, owing to its multiple antibacterial mechanisms and favorable compatibility with biomedical environments.^23–28^

There are two possible mechanisms suggested for the antibacterial activity of ZnO.^20,28^ The first mechanism is the generation of oxygen radicals and hydrogen peroxide (H_2_O_2_) from the defect sites on the ZnO surface.^29–31^ When defect-rich ZnO is activated by UV or visible light, electron–hole pairs (e^–^/h^+^) form. The holes split H_2_O present in the surrounding biological media into OH^–^ and H^+^, while O_2_ is reduced to superoxide radicals (^•^O_2_^–^), which further react to generate H_2_O_2_.^29^ The produced H_2_O_2_ can penetrate bacterial cell membranes and induce cell death.^25,29,32^ The second mechanism is causing damage to the bacterial cell wall due to electrostatic interactions of the dissolved Zn^2+^ ions from the ZnO surfaces.^23,24,27,28,33^

Specifically, previous studies have shown that ZnO is selectively more toxic to both Gram-negative and Gram-positive bacteria compared to healthy human cells,^34,35^ making it a leading choice for an antibacterial coating. Although the antibacterial efficacy of ZnO is relatively lower compared to silver (Ag) and copper (Cu), the cytotoxicity of Zn^2+^ ions is significantly reduced across a wider concentration range,^22,23,26^ making ZnO a safer and more biocompatible alternative for applications in long-term implantable biomedical devices.

Despite their antibacterial potential, ZnO coatings must also demonstrate compatibility with the electrochemical performance requirements of HSR-Pt10Ir electrodes, which are used in neural interfacing applications. While ZnO is moderately conductive and offers advantages such as cost-effective fabrication and broad-spectrum antibacterial efficacy, its electrochemical behavior—particularly when applied to a hierarchically restructured electrode surface—remains largely unexplored. Therefore, this study investigates the electrochemical and microstructural evolution of antibacterial ZnO coatings deposited on HSR-processed Pt10Ir electrodes with the aim of evaluating their viability for dual-function neural interfacing and CRM applications.

In this study, ZnO coatings were deposited via reactive DC magnetron sputtering onto the HSR-Pt10Ir electrodes. Two deposition durations of 5 and 60 min were selected to evaluate the effect of coating thickness on surface and electrochemical properties of ZnO-coated HSR-Pt10Ir electrodes. Comprehensive physical characterization of the ZnO-coated electrodes was conducted to assess their structural, compositional, and morphological features. Electrochemical measurements were performed to evaluate the impact of ZnO deposition on the performance of HSR-Pt10Ir electrodes in a physiological saline solution, simulating the ionic environment of bodily fluids. Antibacterial activity was measured against Gram-positive (*S. aureus*) and Gram-negative (*E. coli*) bacteria. We show that ZnO coatings applied via reactive DC magnetron sputtering to HSR-Pt10Ir electrodes can impart antibacterial functionality to implantable Pt10Ir electrodes while preserving the electrochemical activity essential for long-term clinical performance.

## 3 Materials and Methods

### 3.1 Electrode Fabrication

The electrode fabrication process consisted of two main steps: (i) hierarchical surface restructuring (HSR) of Pt10Ir electrodes and (ii) ZnO layer deposition. The full experimental details of the hierarchical surface restructuring (HSR) process have been discussed in previous reports.^8–10,36^ Briefly, it was performed using a femtosecond laser system (Coherent StarFemto, 1030 nm, 300 fs pulses) under ambient conditions. Flat (unrestructured) Pt10Ir foils (0.3 mm thick) were used as the electrode material and laser-cut into 6 mm diameter discs for electrochemical measurements, 10 mm diameter discs for antibacterial testing, and 10 × 10 mm squares for physical characterization. Surface patterns were generated by using the Visual Laser Marker software integrated with motion and beam control. This process enabled the formation of reproducible micro/nanostructured features, enhancing the surface area and electrode functionality.

A planar DC magnetron sputtering system with a zinc target was employed for reactive sputtering deposition in an oxidizing atmosphere. The deposition utilized an oxygen flow rate of 40 sccm, an argon flow rate of 50 sccm, a sputtering pressure of 4 mTorr, a target-to-substrate spacing of 4 mm, and an RF power of 250 W. All depositions were performed at ambient substrate temperature. The substrates used included planar silicon wafers and HSR-Pt10Ir electrodes. To enhance ZnO film adhesion, a primary zinc layer was deposited in a pure argon atmosphere for 1 min before introducing oxygen for ZnO deposition. ZnO films were deposited for 5 and 60 min to examine representative thin and thick coating regimes and their influence on the electrochemical behavior of HSR-Pt10Ir electrodes. The resulting ZnO-coated HSR-Pt10Ir electrodes are referred to as HSR-Pt10Ir-ZnO-5 and HSR-Pt10Ir-ZnO-60, corresponding to deposition durations of 5 and 60 min, respectively, throughout the paper.

### 3.2 Physical Characterization

X-ray diffraction (XRD) patterns of ZnO films deposited on both silicon wafers and HSR-Pt10Ir electrodes were acquired using a Rigaku Miniflex 600 diffractometer with Cu Kα radiation (λ = 1.5418 Å). Data were collected with a 2θ step size of 0.02° and a scan speed of 1.2° min^–1^. Depth profile analysis via X-ray photoelectron spectroscopy (XPS) was conducted by using a PHI VersaProbe 5000 system with monochromatic Al K_α_ radiation (1486.2 eV) as the X-ray source. Sputter etching was achieved using an Ar^+^ ion gun, with 10 sputtering cycles of 60 s each, followed by spectral acquisition after every cycle. The XPS spot size was set to 200 μm, and calibration was based on the C–C component of the C 1s peak at 285.0 eV. Spectra were analyzed using Casa XPS software. Morphological analysis was carried out using a Zeiss SUPRA50VP scanning electron microscope (SEM) equipped with an in-lens Everhart-Thornley secondary electron detector. SEM micrographs of ZnO-coated HSR-Pt10Ir electrodes were captured at an accelerating voltage of 3 keV and a working distance of 5 mm. To assess the film thickness, a ZnO-coated silicon substrate was cleaved, and cross-sectional imaging was performed using SEM. Milling was performed using a TESCAN S8000X focused ion beam (FIB) microscope equipped with a Xe^+^ ion plasma source. The milling of the HSR-Pt10Ir electrodes was carried out at a FIB current of 100 nA and an accelerating voltage of 30 kV. Elemental analysis and mapping were performed by using an Oxford UltiMax 40-mm^2^ energy dispersive spectrometer (EDS) coupled with Aztec v3.3 software. Measurements of ZnO-coated HSR-Pt10Ir electrodes were conducted at 10 keV, targeting the *M*_α_ lines for Pt and Ir, the *L*_α_ line for Zn, and the *K*_α_ line for O.

### 3.3 Electrochemical Measurements

Cyclic voltammetry (CV) and electrochemical impedance spectroscopy (EIS) measurements were performed using a BioLogic VP3 potentiostat in a custom-designed three-electrode Teflon cell, as used in the previous report.^36^ CV measurements were employed as the primary electrochemical characterization technique to understand the effect of sputter-deposited ZnO coatings on the electrochemical behavior of HSR-Pt10Ir electrodes, including the specific capacitance and charge storage capacity. The obtained specific capacitance and charge storage capacity values directly correlate with the ability of implantable neural interfacing electrodes to store and/or inject charge during pulsing and to efficiently deliver charge to the surrounding tissue,^37^ although CV measurements serve as an accelerated, model characterization rather than a direct representation of in vivo stimulation conditions.^38^ The cell setup included a Ag/AgCl reference electrode (Gamry Instruments Inc., Warminster, PA), a coiled platinum counter electrode, and the working electrodes (HSR-Pt10Ir, HSR-Pt10Ir-ZnO-5, and HSR-Pt10Ir-ZnO-60). A commercially available phosphate-buffered saline (PBS) solution (Blood Bank Saline, Azer Scientific) was used as the electrolyte. The applied voltage was confined to a range that avoids harmful electrochemical reactions at the interface with biological tissue or nerve.^38^ All measurements were performed within a potential range of −0.6 to 0.8 V vs Ag/AgCl at a voltage sweep rate of 50 mV·s^–1^ for 1500 cycles, with all potentials reported vs the Ag/AgCl reference electrode. The electrode–electrolyte contact area of 13.8 mm^2^, calculated from the footprint of the electrode exposed to the electrolyte, was used to determine the specific capacitance and charge storage capacity from the CV data. EIS measurements were performed at the open circuit potential (OCP) over a frequency range of 0.1–10^5^ Hz, using a sinusoidal excitation voltage of 10 mV (*V*_rms_), for all the working electrodes after 15, 30, and 1500 CV cycles.

### 3.4 Antibacterial Measurements

*Escherichia coli* K-12 (Gram-negative) and *Staphylococcus aureus* ATCC 27660 (Gram-positive) were used as representative bacterial strains to assess bactericidal efficacy. Active bactericidal species (e.g., Zn^2+^ ions) from a coated electrode can diffuse into the bacteria-containing media on top of the agar. When the bactericidal species’ release exceeds a critical threshold, bacterial growth is inhibited in the associated media, resulting in a zone of inhibition (ZOI) of bacterial growth around the electrode, which appears as a transparent circle on an otherwise opaque agar plate that is covered by growing bacterial cells outside of the ZOI. The antibacterial performance of the electrode surfaces was investigated using a modified Kirby–Bauer ZOI assay.^39^ In this adapted procedure, electrodes were autoclaved at 121 °C for 30 minutes and aseptically placed with the coated surface oriented downward on top of solidified agar (VWR Life Science, Agarose RA, 7.5 g/L) containing 14 g/L Luria Broth (LB; RPI Research Products, L24080) in a Petri dish. Electrodes were incubated on the agar surface for 2 h at room temperature to facilitate electrode–agar interactions and ion diffusion from the electrode into agar. Subsequently, liquid samples of log-phase bacterial cells (0.5–2 × 10^6^ CFU/plate) were spread across the agar plates and around electrodes, which were then incubated under completely dark, aerobic conditions at 37 °C for 18 h. Specifically, the glass door on the incubator was covered with aluminum foil to ensure darkness and to study the bactericidal efficacy of the electrodes under simulated implanted/dark conditions. The concentration of colony-forming units (CFU/mL) in each experiment was determined in parallel cultures by diluting the initial culture 10 million-fold (i.e., *D* = 10^7^) in LB and then spreading 1 mL of those cells onto agar plates, which were incubated overnight at 37 °C for 18 h alongside the plates with the electrodes. The number of colonies on each plate (*N*) were then counted, allowing us to calculate the CFU concentration of the initial culture using eq 1.

1 $$\mathrm{CFU}/\mathrm{mL}=D^{*}N/1\mathrm{mL}$$

Following incubation, the antimicrobial efficacy of each coating was quantified by digitally measuring the width of the ZOI based on the corresponding electrode disc’s known diameter. Due to the small deviations in the ZOI width between each experiment, three independent biological replicates were conducted for each type of electrode.

## 4 Results and Discussion

The XRD pattern of HSR-Pt10Ir-ZnO-60 (Figure 2) shows distinct characteristic peaks of the crystalline ZnO phase. The major peaks observed at 32.07°, 34.47°, and 36.53° values of 2θ correspond to the (100), (002), and (101) crystallographic planes of ZnO, respectively. The grazing incidence XRD pattern of silicon substrate coated with ZnO for 60 min deposition (Figure S1 in the Supporting Information) shows major peaks at 34.8 and 64.5° values of 2θ corresponding to the (002) and (103) crystallographic planes of ZnO, respectively. All the ZnO-coated substrates exhibit diffraction peaks only corresponding to ZnO, indicating the absence of secondary Zn-based phases such as metallic Zn. The substrates coated for 5 min did not exhibit ZnO peak, likely due to insufficient film thickness. Cross-sectional SEM micrographs of a ZnO film deposited on a silicon substrate for 1 h (Figure S2 in Supporting Information) revealed a thickness of approximately 280–300 nm, corresponding to a film growth rate of ∼4.5 nm·min^–1^. Based on this rate, the thickness of ZnO films deposited on silicon for 5 min is estimated to be around 25–30 nm.

**Figure 2:**
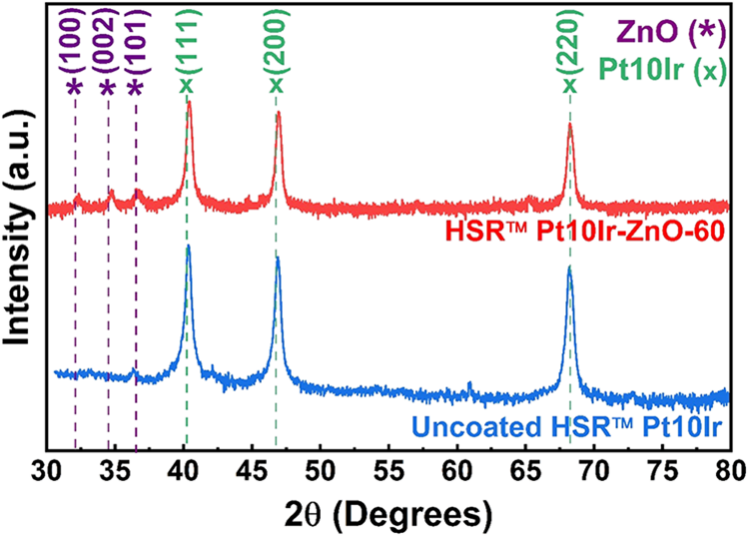
XRD patterns of uncoated HSR-Pt10Ir (blue-bottom) and HSR-Pt10Ir-ZnO-60 (red-top) electrodes. ZnO peaks are marked with purple dashed lines and an (*) symbol, and Pt10Ir peaks are indicated by green dashed lines and an (x) symbol.

To further confirm the presence of ZnO and better understand the characteristics of the sputtered ZnO films, XPS analysis was performed. Figure 3 presents the XPS spectra of the ZnO-coated HSR-Pt10Ir electrodes. The survey scans of the ZnO-coated HSR-Pt10Ir electrodes revealed characteristic peaks corresponding to Zn 2p and O 1s, confirming the formation of ZnO. For both HSR-Pt10Ir-ZnO-5 (Figure 3a) and HSR-Pt10Ir-ZnO-60 (Figure 3b), the Zn 2p peak splits into Zn 2p_3/2_ and Zn 2p_1/2_ due to spin–orbit interaction, with a doublet peak energy separation of approximately 23.0 eV. In case of both HSR-Pt10Ir-ZnO-5 and HSR-Pt10IrZnO-60, the Zn 2p_3/2_ peak was observed at 1021.6 eV and the Zn 2p_1/2_ peak at 1044.5 eV. These values are well within the typical range of binding energies reported for ZnO.^40–42^ Figure 3c,3d present the O 1s spectra for HSR-Pt10Ir-ZnO-5 and HSR-Pt10Ir-ZnO-60, respectively. For HSR-Pt10Ir-ZnO-5 (Figure 3c), the deconvoluted peaks were identified at 530.37 and 531.87 eV, corresponding to O­(I) and O­(II), respectively. The lower binding energy peak O­(I), centered at 530.37 eV, is attributed to lattice oxygen,^43,44^ which contributes to the hexagonal wurtzite structure of the ZnO lattice. The higher binding energy peak O­(II), centered at 531.87 eV, has recently been attributed to oxygen from chemisorbed water molecules.^44^ For HSR-Pt10Ir-ZnO-60 (Figure 3d), the deconvoluted peaks of the O 1s peaks were observed at 530.41 eV for O­(I) and 531.94 eV for O­(II).

**Figure 3:**
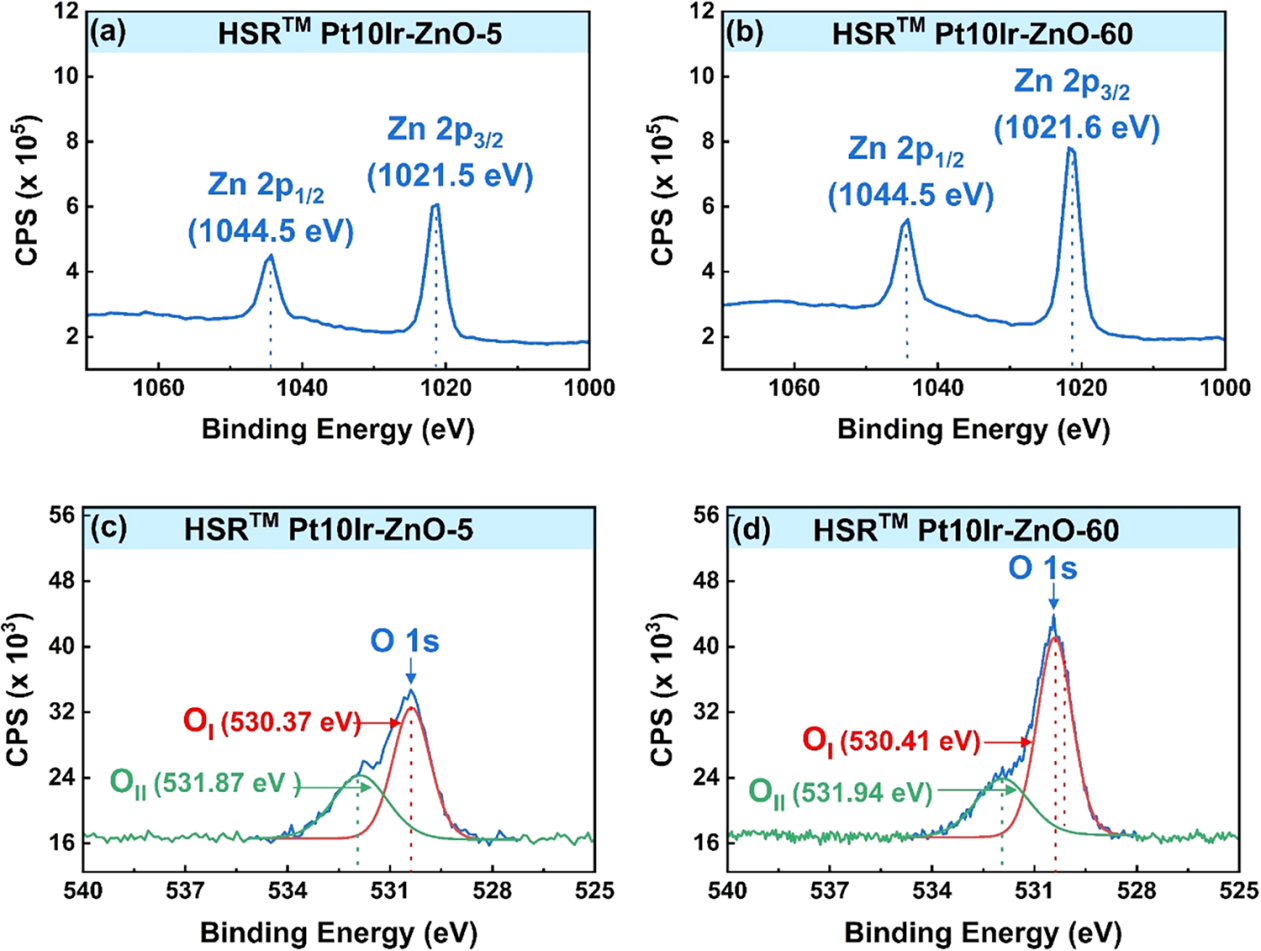
XPS spectra of HSR-Pt10Ir-ZnO-5 for (a) zinc 2p, (c) oxygen 1s, and HSR-Pt10Ir-ZnO-60 for (b) zinc 2p, (d) oxygen 1s. Deconvoluted peaks for the oxygen spectra are shown with red and green lines.

Figure 4a–c shows SEM micrographs of uncoated HSR-Pt10Ir electrodes at increasing magnifications. The surface of the uncoated HSR-Pt10Ir electrode exhibits a hierarchical topography composed of periodic mound-like pillars with diameters of ∼20–25 μm and a valley depth of 25 to 30 μm. These pillars are covered by a finer nanoscale structure, with features on the order of a few hundred nanometers in size that contribute to the overall surface roughness. This roughness originates from femtosecond laser restructuring and can be tuned by varying specific laser parameters, such as average power and fluence.^8,10^ Figure 4d–f shows SEM micrographs of HSR-Pt10Ir-ZnO-5 electrodes, while Figure 4g–i displays SEM micrographs of HSR-Pt10Ir-ZnO-60 electrodes. Comparison of the low-magnification SEM micrographs of uncoated electrodes with those of sputter-coated electrodes (top row of Figure 4) reveals that the PVD coating process does not significantly alter the overall hierarchical surface morphology. At higher magnifications, the surface of the uncoated HSR-Pt10Ir pillars (Figure 4c) exhibits secondary features such as pores and surface roughness. After ZnO deposition, as observed in Figure 4f,i, the coating appears as agglomerated nanospheres with diameters ranging from 40 to 60 nm.

**Figure 4:**
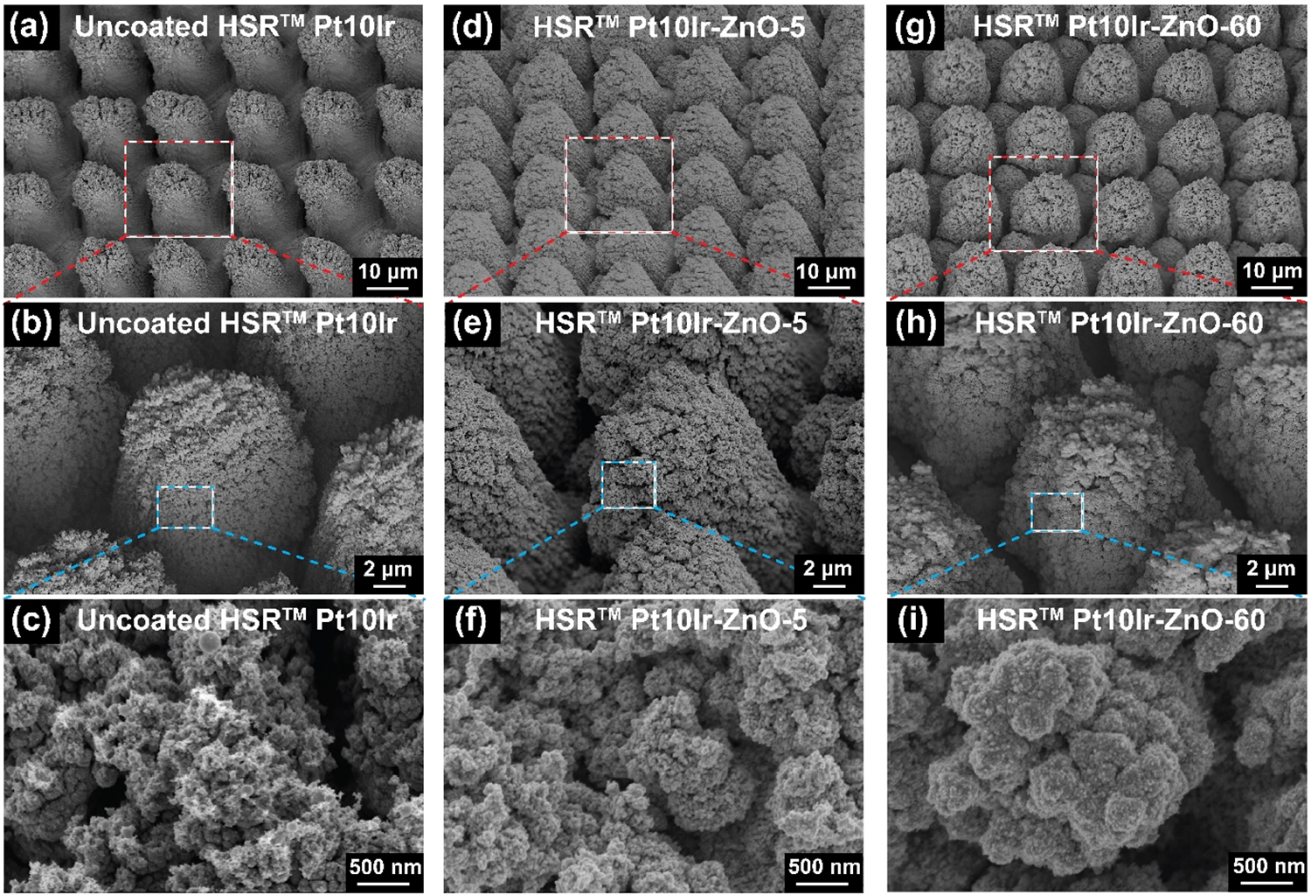
SEM micrographs of uncoated HSR-Pt10Ir (a–c), HSR-Pt10Ir-ZnO-5 (d–f), and HSR-Pt10Ir-ZnO-60 (g–i) electrodes. Micrographs (a, d, g) display the micropillar array architecture of the electrodes, micrographs (b, e, h) focus on the morphology of a single pillar, and the higher magnification micrographs (c, f, i) reveal the nanostructured morphology of the ZnO coating over the micropillars.

Figure 5 shows the cyclic voltammograms of the electrochemical cells containing HSR-Pt10Ir, HSR-Pt10Ir-ZnO-5, and HSR-Pt10Ir-ZnO-60 electrodes, corresponding to the second, 15th, 30th, and 1500th cycles. The CV curves of HSR-Pt10Ir-ZnO-5 (Figure 5b) and HSR-Pt10Ir-ZnO-60 (Figure 5c) electrodes exhibit semirectangular shapes similar to the uncoated HSR-Pt10Ir electrode (Figure 5a), indicating that the capacitive behavior is retained after ZnO deposition. However, the area under the CV curves increased notably following ZnO deposition. As a result, the specific capacitance, calculated from the second CV cycle, increased by 105 μF·mm^–2^ for HSR-Pt10Ir-ZnO-5 (506.1 μF·mm^–2^) and 140 μF.mm^–2^ for HSR-Pt10Ir-ZnO-60 (541.3 μF·mm^–2^) electrodes compared to the uncoated HSR-Pt10Ir (400.9 μF·mm^–2^) electrode. This increase in the specific capacitance confirms the presence of a new material on the coated electrode surface. Despite the assumption that ZnO’s moderately conductive nature^45^ might diminish the performance of HSR-Pt10Ir electrodes, the specific capacitance of both HSR-Pt10Ir-ZnO-5 and HSR-Pt10Ir-ZnO-60 electrodes was higher than that of uncoated ones. To understand this enhancement, the conformality of sputtered ZnO film over the HSR topography was examined, as capacitance is largely governed by the material’s electrochemically active surface area (ESA).

**Figure 5:**
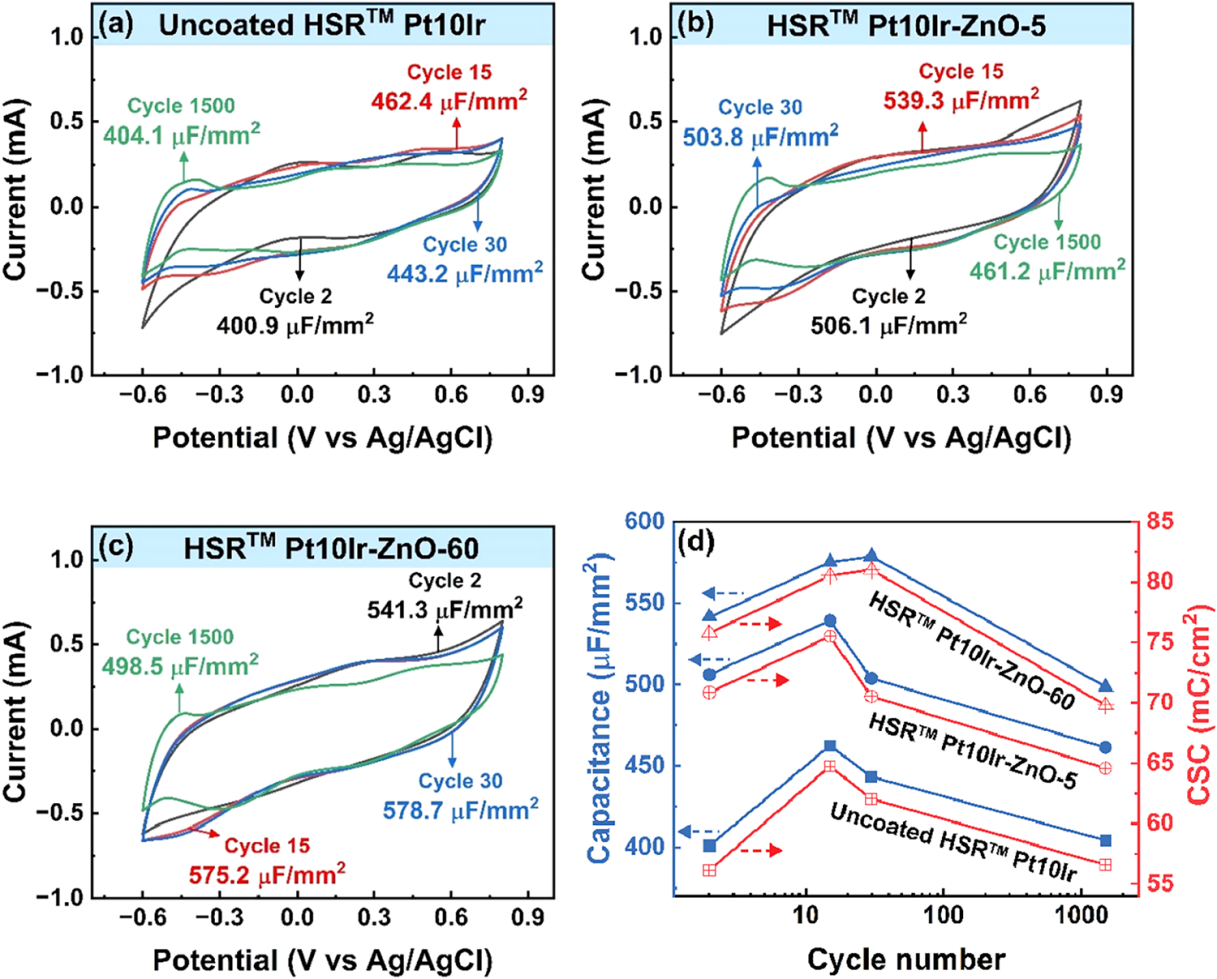
Cyclic voltammograms of (a) uncoated HSR-Pt10Ir, (b) HSR-Pt10Ir-ZnO-5, and (c) HSR-Pt10Ir-ZnO-60 electrodes measured at a scan rate of 50 mV·s^–1^. Specific capacitance values at each cycle are indicated on the respective plots. (d) Evolution of specific capacitance (left *y*-axis) and charge storage capacity (right *y*-axis) as a function of CV cycle number (log scale) for all three electrodes: uncoated HSR-Pt10Ir (squares), HSR-Pt10IrZnO-5 (circles), and HSR-Pt10Ir-ZnO-60 (triangles).

The conformality of the sputtered ZnO films was examined by using FIB cross-sectioning and EDS mapping, as shown in Figure 6. Selected pillars from the HSR-Pt10Ir-ZnO-5 electrode were milled to expose the lateral surfaces of the adjacent pillars. Subsequent EDS mapping of the area (Figure 6c,d) confirmed the presence of zinc and oxygen on the HSR-Pt10Ir surface. The analysis revealed that while the tops of the pillars were predominantly coated with ZnO, the sides exhibited only minimal coverage. This indicates that the sputtered ZnO films lack conformality and do not evenly coat the pillar-like structures. Instead, ZnO primarily increases the height of the pillars without providing uniform coverage. This uneven film distribution effectively increases the active ESA within the same footprint, contributing to higher capacitance values.

**Figure 6:**
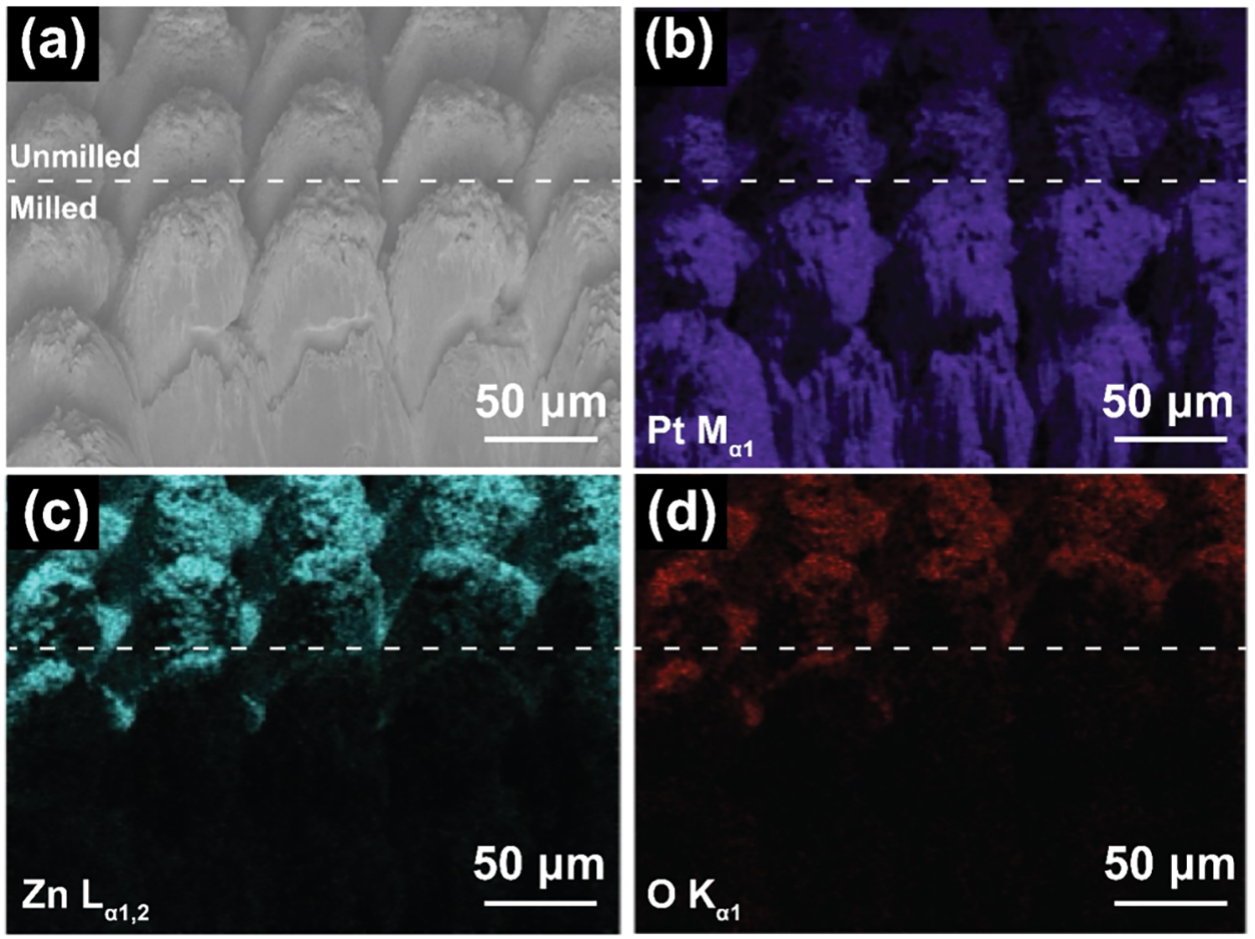
SEM micrographs and elemental EDS mapping of the HSR-Pt10Ir-ZnO-5 surface after FIB cross-sectioning. (a) SEM micrograph; (b) EDS mapping of platinum; (c) EDS mapping of zinc; and (d) EDS mapping of oxygen.

Additionally, the partial ZnO coating leaves some regions of the Pt10Ir pillars exposed and uncoated. This observation prompted further investigation of the nature of these uncoated areas. Since the ZnO deposition occurs in an oxidizing environment inside the sputtering chamber, there is a possibility that the Ir in the HSR-Pt10Ir substrate may oxidize, given the high affinity of Ir toward oxidation. A mock sputtering experiment was conducted to investigate this phenomenon. The HSR Pt10Ir substrate was placed inside the sputtering chamber under the same conditions used for ZnO deposition, except that the substrate stage was oriented away from the Zn target to prevent any ZnO coating. After 5 min, the substrate (now referred to as oxidized HSR-Pt10Ir) was removed, and depth-profile XPS analysis was performed to determine whether Ir oxidation had occurred and penetrated beneath the surface. The CV results and XPS spectra of Ir 4f after 2 min of surface etching obtained from oxidized HSR-Pt10Ir electrode are shown in Figure S3a,b in the Supporting Information, respectively. Following peak fitting,^46^ the binding energies and relative area percentages of the deconvoluted peaks are summarized in Table S1 in Supporting Information. An increase in the relative area associated with the Ir^4+^ species was observed after the mock oxidation experiment. Hence, it was confirmed that the exposed uncoated regions of the ZnO-coated HSR-Pt10Ir contain oxidized Ir. Due to the nonconformal nature of the sputtered ZnO coating, certain parts of the HSR surface remain exposed without ZnO coating, allowing the partially oxidized Pt10Ir substrate to be in contact with the electrolyte. The presence of exposed conductive regions supports efficient electron transport despite the moderate conductivity of the ZnO coating. However, surface Ir oxidation is not considered to be the primary origin of the observed capacitance enhancement. While oxidized Ir in the partially exposed Pt10Ir metallic surface of the electrode may provide a secondary contribution to the electrochemical response, the dominant mechanism governing the increased specific capacitance of the HSR-Pt10Ir-ZnO-5 and HSR-Pt10Ir-ZnO-60 electrodes is likely the increase in electrochemically active surface area resulting from the nonconformal ZnO coating.

A schematic illustration of the nonconformal growth of sputtered ZnO films on the HSR-Pt10Ir electrode surface is presented in Figure 7. As shown in Figure 7b,7c, ZnO deposition increases the height of the micropillars with longer deposition times, leading to a higher ESA within the same geometric surface area. Due to the nonconformal nature of the ZnO deposition, the regions along the micropillar sidewalls remain uncoated, leaving the underlying Pt10Ir surface uncoated and, thus, partially oxidized.

**Figure 7:**
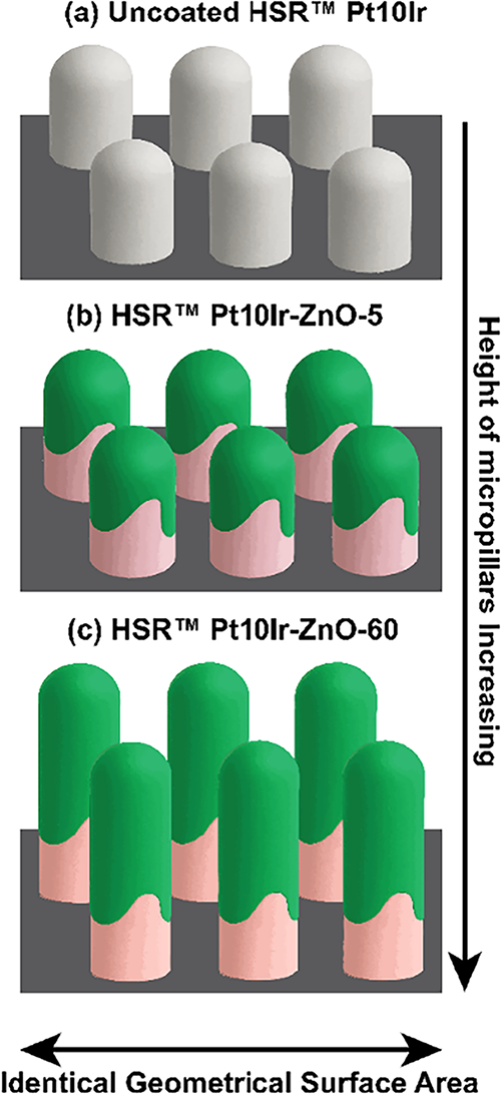
Schematic illustration showing the growth and nonuniform coverage of the ZnO coating (green) on HSR-Pt10Ir electrodes along with Ir oxidation (pink) induced during sputtering: (a) uncoated HSR Pt10Ir, (b) HSR-Pt10Ir-ZnO-5, and (c) HSR-Pt10Ir-ZnO-60 electrodes. The change in the pillar height is shown for illustration purposes only and is not drawn to scale.

Electrochemical impedance spectroscopy (EIS) was used to investigate the evolution of the HSR-Pt10Ir-ZnO-5 and HSR-Pt10Ir-ZnO-60 electrodes during prolonged cycling. Bode impedance plots of the HSR-Pt10Ir, HSR-Pt10Ir-ZnO-5, and HSR-Pt10Ir-ZnO-60 electrodes after 15, 30, and 1500 CV cycles are shown in Figure 8a–8c, respectively. The analysis indicates that after 15 CV cycles (Figure 8a), the impedance of the HSR-Pt10Ir-ZnO-5 and HSR-Pt10Ir-ZnO-60 electrodes is notably higher than that of the uncoated HSR-Pt10Ir electrode. The increased impedance observed in HSR-Pt10Ir-ZnO-5 and HSR-Pt10Ir-ZnO-60 electrodes during initial cycling is attributed to the ZnO coating, which introduces additional resistance to electron transport due to its lower conductivity compared with the metallic Pt10Ir. However, after 30 cycles (Figure 8b), the impedance of the HSR-Pt10Ir-ZnO-5 electrode decreases and approaches the value of the uncoated HSR-Pt10Ir electrode. Similarly, after 1500 cycles (Figure 8c), the impedances of both HSR-Pt10Ir-ZnO-5 and HSR-Pt10Ir-ZnO-60 electrodes decrease and match the value of the uncoated electrode. These observations suggest a gradual removal of the ZnO layer, likely due to the dissolution in electrolyte, which exposes more of the conductive Pt10Ir surface and reduces the overall resistivity, leading to improved electron transport and lower impedance as observed in the EIS Bode plots.

**Figure 8:**
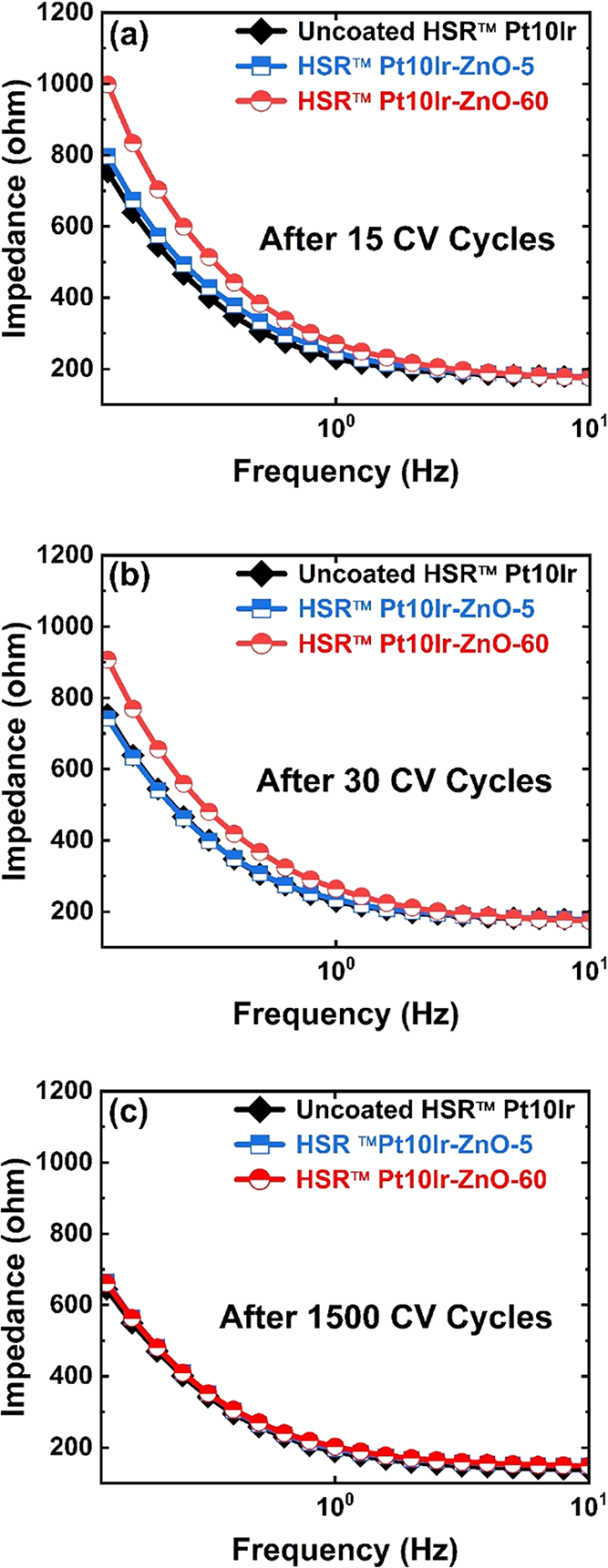
Bode impedance plots of uncoated HSR-Pt10Ir (black), HSR-Pt10Ir-ZnO-5 (blue), and HSR-Pt10Ir-ZnO-60 (red), measured after (a) 15, (b) 30, and (c) 1500 CV cycles.

The gradual stripping of ZnO during CV cycling was further investigated using postcycling SEM and EDS analyses. Figure 9 shows the postcycling SEM micrographs and EDS mapping of HSR-Pt10Ir-ZnO-5 and HSR-Pt10Ir-ZnO-60 electrodes. The cycling was stopped after 1500 CV cycles (approximately 24 h of electrochemical cycling) at −0.6 V. Post-CV EDS analysis confirms that following 1500 CV cycles, the sputtered ZnO layer is no longer detectable on the electrode surface for either HSR-Pt10Ir-ZnO-5 (Figure 9a–d) or HSR-Pt10Ir-ZnO-60 (Figure 9e–h) electrodes. This observation indicates that, under the applied potential window used during continuous CV cycling, the sputtered ZnO coating progressively dissolves into the electrolyte, a process that is integral to the antibacterial functionality of ZnO in the surrounding medium. ZnO is widely reported^23,28^ to exhibit antibacterial activity through the release and diffusion of Zn^2+^ ions into the environment, where these ions can disrupt bacterial cell walls and interfere with intracellular processes, ultimately resulting in bactericidal effects.

**Figure 9:**
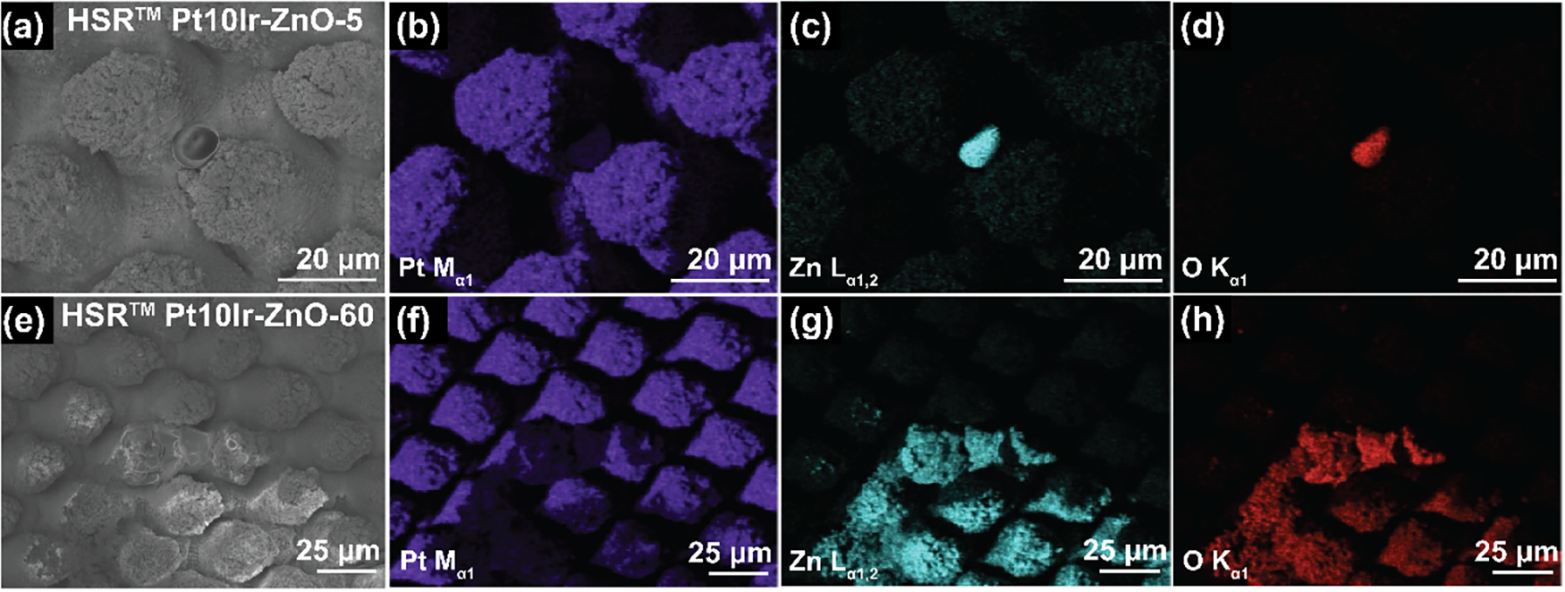
SEM micrographs and EDS mapping of (a)–(d) HSR-Pt10Ir-ZnO-5 electrode, and (e)–(h) HSR-Pt10Ir-ZnO-60 electrode, after 1500 CV cycles (24 h extended cycling) stopped at −0.6 V.

The ZnO dissolution behavior observed during cyclic voltammetry is governed by the electrochemical conditions imposed by continuous cycling across a wide potential window. In practical device operation, implantable electrodes are not subjected to such sustained polarization but instead operate under low-amplitude, charge-balanced pulsed voltage, or current stimulation. Under these clinically relevant regimes, the electrochemical driving forces that govern ZnO dissolution are expected to differ substantially, resulting in a dissolution behavior that more closely reflects long-term in vivo conditions.

In addition to ZnO dissolution, as a consequence of the confined electrolyte volume during CV measurements, localized Zn- and O-rich features (Figure 9c–g,h) were also observed after 1500 CV cycles, indicating ZnO recrystallization at isolated surface sites. While ZnO recrystallization is typically reported at more negative potentials (−0.8 to −2 V vs Ag/AgCl),^47–49^ the hierarchical surface geometry of the HSR-Pt10Ir electrodes may give rise to spatially heterogeneous electrochemical environments,^50–52^ which could facilitate limited, site-specific redeposition even at higher applied potentials such as the ones used in our experiments.

The observed ZnO recrystallization also serves as indirect evidence of Zn^2+^ ion release into the electrolyte, which is a well-established antibacterial mechanism for ZnO. Furthermore, the occurrence of localized recrystallization likely suggests that, under the specific CV conditions, the local electrolyte environment became enriched with Zn^2+^ ions, indicating a high amount of ZnO mass loading on the HSR-Pt10Ir electrode surface that can support its antibacterial functionality. During device-specific stimulation pulses, the dissolution of Zn^2+^ ions is expected to proceed at a different rate, and the released Zn^2+^ ions into the surrounding environment will likely be consumed through antibacterial interactions or diffuse away from the electrode surface into the bloodstream. Under such conditions, the accumulation of Zn species required for recrystallization is unlikely, and ZnO dissolution will likely remain the dominant and functionally relevant process.

Based on the CV cycling and postcycling characterization of HSR-Pt10Ir-ZnO electrodes presented above, we propose the following mechanism for the evolution of ZnO coatings during prolonged electrochemical cycling: when exposed to the saline solution electrolyte during CV cycling, HSR-Pt10Ir-ZnO-5 and HSR-Pt10Ir-ZnO-60 electrodes exhibit higher capacitances than the uncoated HSR-Pt10Ir electrode, primarily due to the increased ESA resulting from the sputtered, nonconformal ZnO layer. Additionally, the increased capacitance can be attributed to the partially oxidized Ir in the composition of the exposed uncoated regions of the HSR-Pt10Ir electrode. Despite the moderate conductivity of the ZnO film, the overall electron transport remains efficient, supported by the underlying and uncoated metallic Pt10Ir regions of the electrode surface. As cycling progresses, the deposited ZnO layer gradually strips from the electrode surface and dissolves into the electrolyte, consistent with the observed decrease in impedance of the ZnO-coated electrodes over extended electrochemical cycling. ZnO dissolution was further confirmed through postcycling EDS mapping of the ZnO-coated HSR-Pt10Ir electrodes. After extended cycling, SEM micrographs and EDS mapping revealed localized recrystallization of ZnO on the electrode surface, forming well-faceted crystals. The agglomeration of bulky recrystallized ZnO particles reduces the available ESA, leading to a decrease in the specific capacitance at later cycles (i.e., cycle 1500). Notably, the HSR-Pt10Ir-ZnO-60 electrode, with a thicker ZnO layer, experiences prolonged ZnO dissolution that delays recrystallization, thereby better preserving the capacitance during extended cycling.

While the electrochemical measurements in this study were carried out in a confined environment with continuous voltage application during CV experiments which allowed the recrystallization of dissolved ZnO on the electrode surface, in practical implantable applications, the electrodes will be subjected to pulsed currents or voltages within a larger biological environment. Under these conditions, the dissolved Zn^2+^ ions are expected to interact with and diffuse into the bacterial cell walls to eliminate them.^28^ This leads to subsequent consumption of Zn^2+^ ions, therefore, preventing ZnO recrystallization on the electrode surface.

To verify the antibacterial activity of the electrodes, a modified Kirby–Bauer zone of inhibition (ZOI) method was used to test the ZnO-coated HSR-Pt10Ir electrodes against *S. aureus* (Gram-positive) and *E. coli* (Gram-negative) bacteria. The HSR-Pt10Ir-ZnO-60 electrode was selected for this study as it has a higher ZnO mass loading and demonstrated the best electrochemical performance. Figure 10 shows that the HSR-Pt10Ir-ZnO-60 electrode exhibits a clear ZOI as compared to uncoated HSR-Pt10Ir electrodes against both *S. aureus* (Figure 10a) and *E. coli* (Figure 10b) bacterial strains. The average ZOI width observed for *E. coli* was 3.93 ± 0.46 mm, while the ZOI for *S. aureus* was 2.10 ± 0.42 mm, indicating that ZnO has higher bactericidal efficacy for *the Gram-negative E. coli* compared to the Gram-positive *S. aureus*.

**Figure 10:**
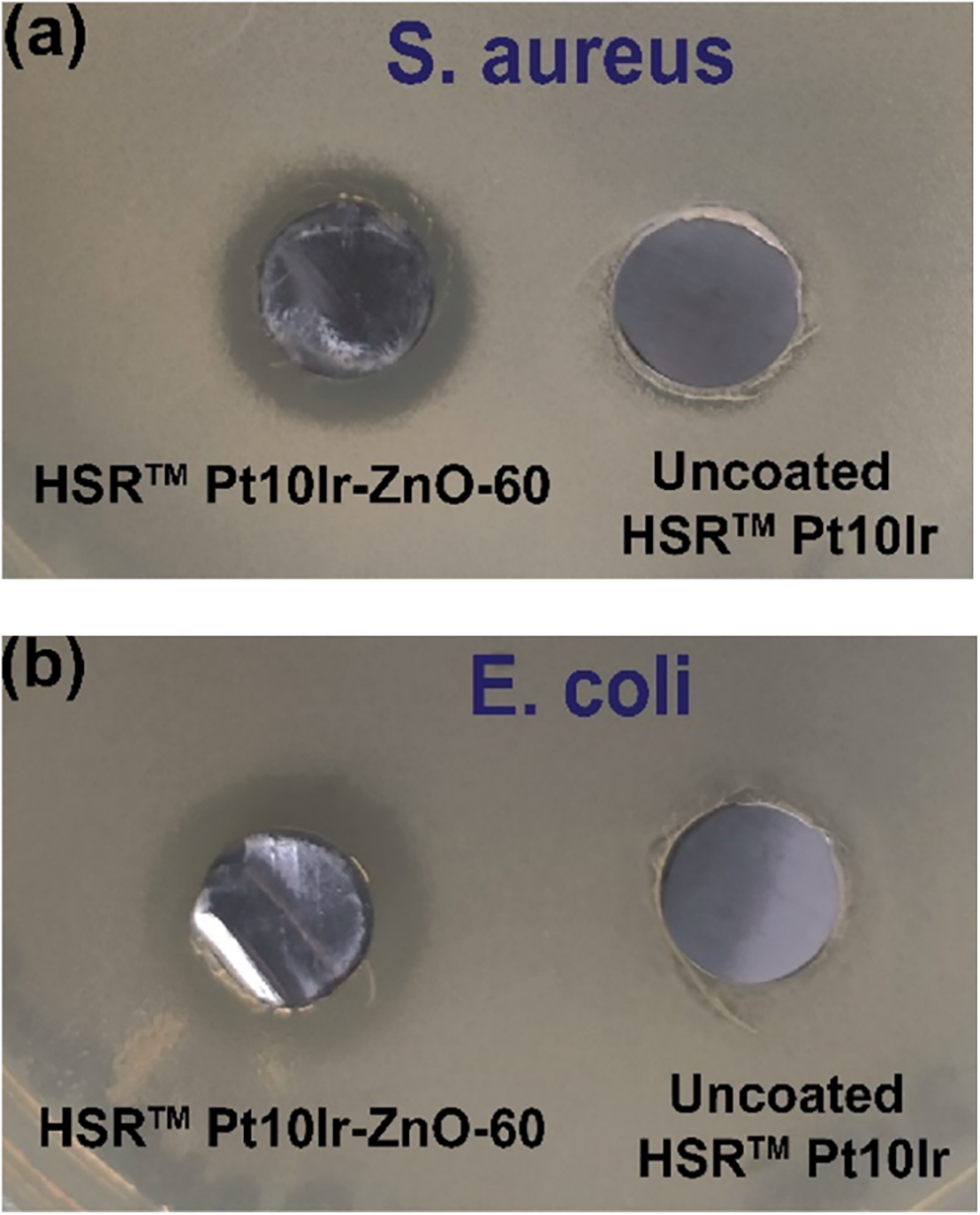
Antibacterial activity of HSR-Pt10Ir-ZnO-60 electrode alongside uncoated HSR-Pt10Ir electrode against (a) *S. aureus* and (b) *E. coli* bacterial strains. ZOI is clearly visible for HSR-Pt10Ir-ZnO-60 electrodes as a ring of different contrast around them. The electrodes are 10 mm discs, which can be used as scale bars to measure ZoI.

The inhibition of bacterial growth extending beyond the ZnO-coated surfaces indicates that antimicrobial species diffuse from the coating into the surrounding medium. As all experiments were conducted under dark conditions, the active species are most likely Zn^2+^ ions released from the ZnO layer. This ion release appears sufficient to achieve effective inhibition of both bacterial strains, likely through Zn^2+^-mediated disruption of cell membrane integrity and associated impairment of cellular function. These findings demonstrate that sputter-deposited ZnO coatings can reliably endow HSR-Pt10Ir electrodes with antibacterial functionality.

## 5 Concluding Remarks

This study establishes a dual-functional electrode platform in which reactively sputtered zinc oxide coatings, integrated with hierarchically restructured platinum–iridium (Pt10Ir) electrodes, synergistically enhance electrochemical performance while imparting robust antibacterial functionality. Deposition of ZnO across the hierarchically restructured Pt10Ir electrode surface increases the electrochemically active surface area (ESA), resulting in specific capacitance enhancements of approximately 16% and 24% for the HSR-Pt10Ir-ZnO-5 (at ∼30 nm ZnO) and HSR-Pt10Ir-ZnO-60 (at ∼300 nm ZnO) electrodes, respectively, relative to uncoated HSR-Pt10Ir control electrodes. X-ray photoelectron spectroscopy confirms the formation of oxidized Ir species within partially exposed micropillar regions, indicating that controlled surface oxidation facilitates efficient charge transport and partially contributes to the observed capacitance enhancement.

Electrochemical impedance spectroscopy reveals a progressive decrease in the interfacial impedance during extended cycling, consistent with gradual ZnO dissolution into the electrolyte. Following prolonged cyclic voltammetry experiments, localized ZnO recrystallization is also observed on the electrode surface. However, continuous CV cycling over wide potential windows does not reflect the practical device operation. Implantable neurostimulation and cardiac rhythm management electrodes are engineered for long-term stimulation using low-amplitude, charge-balanced, constant-current pulsed waveforms, under which the electrochemical conditions that promote ZnO dissolution and recrystallization during extended CV testing are unlikely to arise. The dissolution and stability behavior observed under prolonged CV cycling should therefore be interpreted as an accelerated electrochemical stress condition rather than a direct analogue of in vivo performance.

Taken together, the results of this study provide a foundational demonstration of integrating sputter-deposited, cost-effective, and commercially viable ZnO coatings with HSR-Pt10Ir electrodes. The observed antibacterial activity confirms that ZnO-coated HSR-Pt10Ir electrodes can effectively suppress bacterial proliferation while preserving and, in some cases, enhancing electrochemical performance. Collectively, these findings identify sputter-deposited ZnO as a promising materials integration strategy for implantable neurostimulation and cardiac rhythm management applications.

Future work will focus on long-term stimulation studies under physiologically relevant conditions using pulsed electrical signals and simulated body fluids to further assess coating stability, sustained antibacterial activity, and overall electrode performance.

## Supplementary Material



## Notes

XRD pattern of Si wafer coated with ZnO for a 60 min deposition duration, cross-sectional SEM micrograph of cleaved ZnO-coated planar Si wafer deposited for 60 min, CV cycling results of oxidized HSR-Pt10Ir electrode, XPS spectra, and peak fitting analysis of oxidized HSR-Pt10Ir electrode (PDF)
