# Supplementary material for: Hierarchically Restructured Antibacterial Electrodes for Neural Interfaces: Electrochemical and Microstructural Evolution under Extended Cycling
Source: ACS Appl Mater Interfaces. 2026 Mar 16;18(11):16033–44. doi: 10.1021/acsami.5c21727 (PMC13022803; doi:10.1021/acsami.5c21727)
Supplement: Supplementary file 1 [file am5c21727_si_001.pdf]

Supporting Information

Hierarchically Restructured Antibacterial Electrodes for Neural Interfaces: Electrochemical and Microstructural Evolution Under Extended Cycling

Kriti Panchal [1], Wesley Seche [2], Henna Khosla [3], Gang Feng [3], Jacob Elmer [4], Gregory A. Caputo [6], Steven J. May [1], Ekaterina Pomerantseva [*1], Shahram Amini [*2,5]

[1] Department of Materials Science and Engineering, Drexel University, Philadelphia, PA 19104 USA

[2] Pulse Technologies Inc. (An Integer Holdings Company), Quakertown, PA 18951 USA

[3] Department of Mechanical Engineering, Villanova University, Villanova, PA 19085 USA

[4] Department of Chemical and Biological Engineering, Villanova University, Villanova, PA 19085 USA

[5] Biomedical Engineering Department, University of Connecticut, Storrs, CT 06269 USA

[6] Department of Chemistry and Biochemistry, Rowan University, Glassboro, NJ 08028 USA

***Co-corresponding authors:** Correspondence should be addressed to:

SA (email: shahram.amini@integer.net) and EP (email: ep423@drexel.edu)

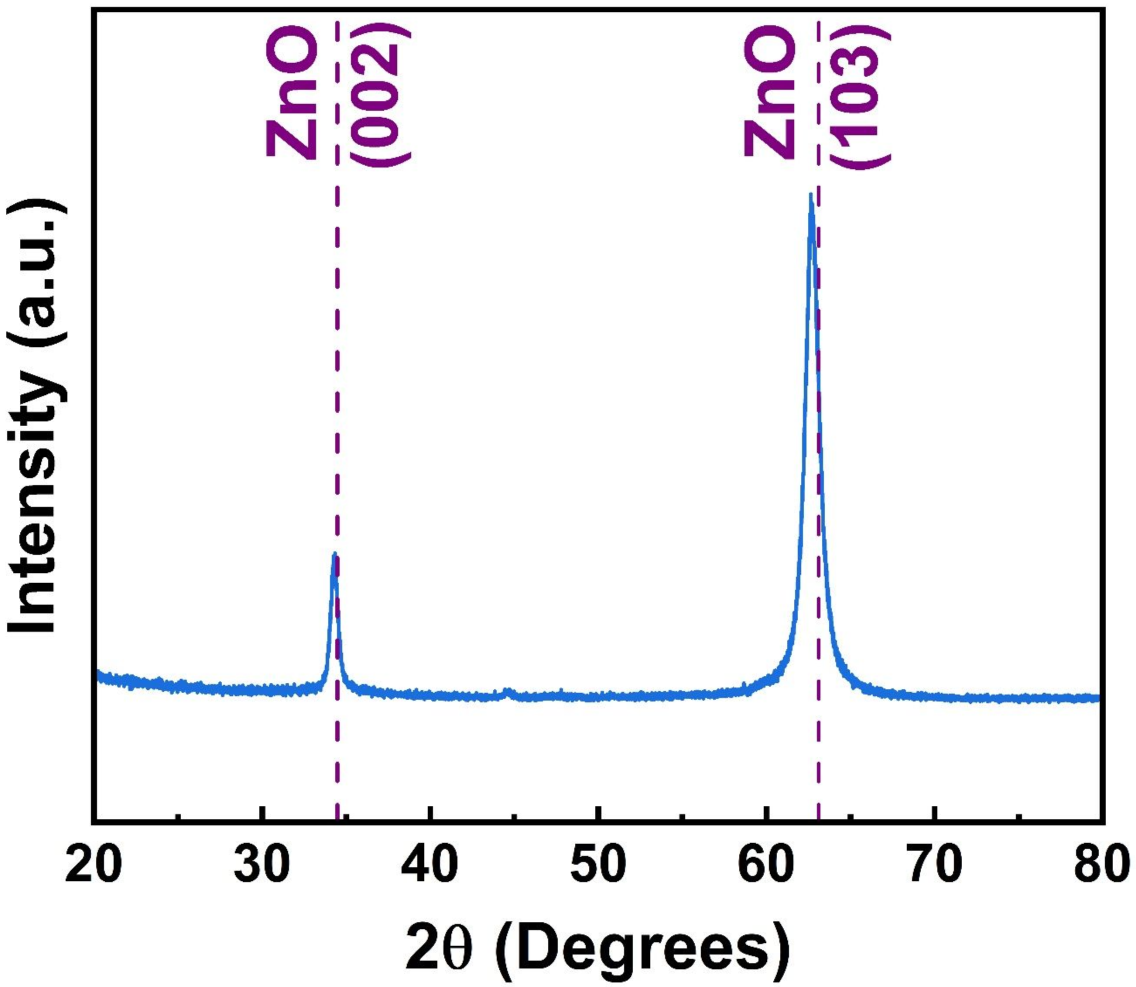

Figure S1: XRD pattern of silicon wafer coated with ZnO for a 60-minute deposition duration. ZnO (002) and (103) peaks are indicated by purple dashed lines.

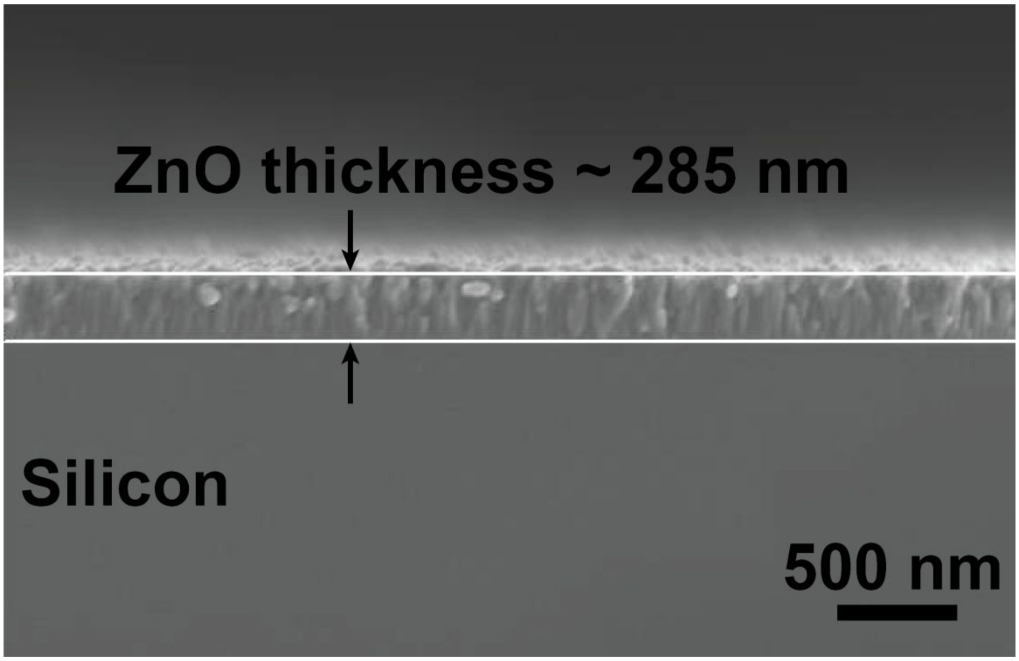

Figure S2: Cross-sectional SEM micrograph of cleaved ZnO-coated planar Si wafer deposited for 60 minutes used to determine the ZnO growth rate.

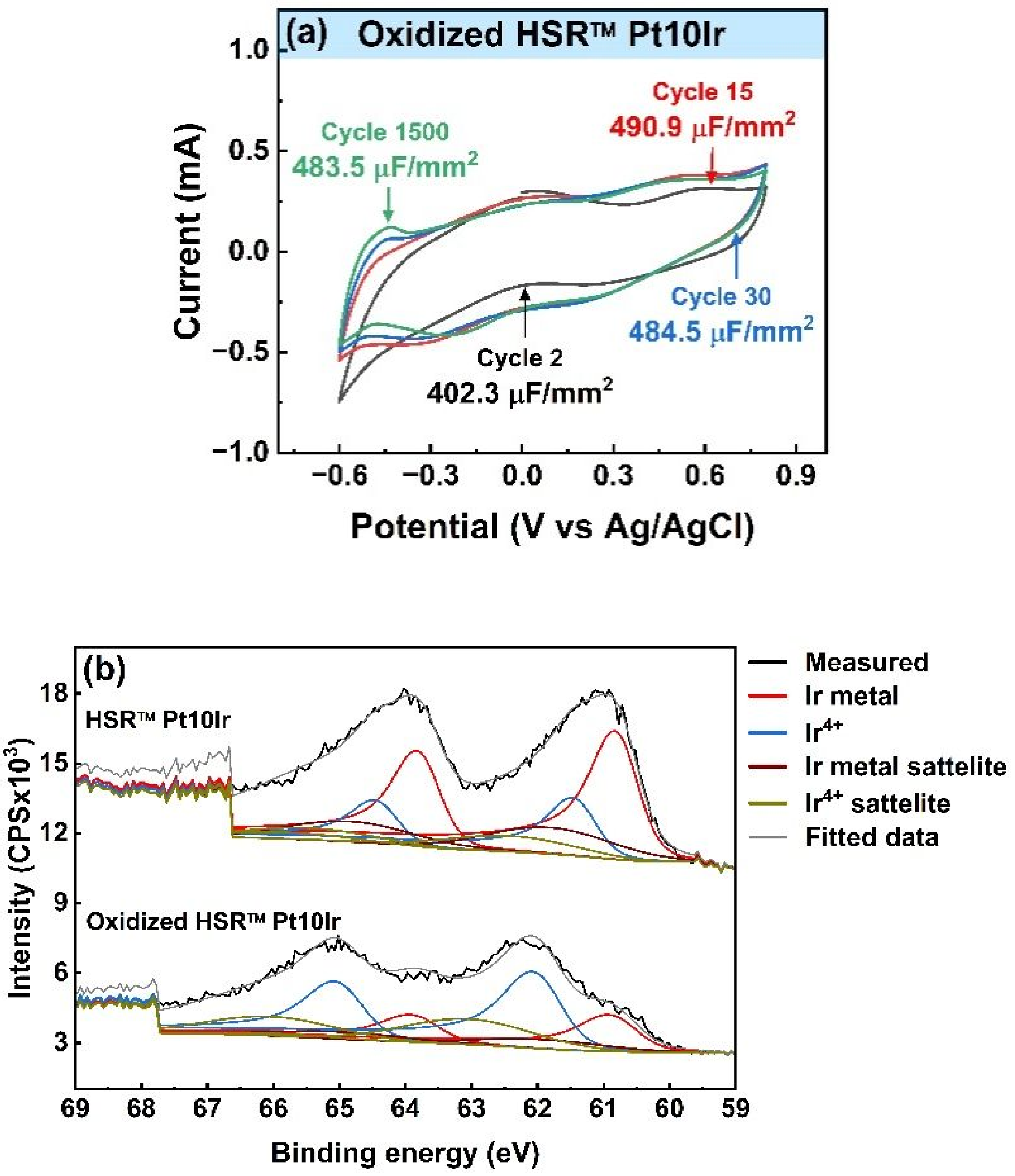

Figure S3: (a) CV curves of oxidized HSR™ Pt10Ir electrode after mock-oxidation experiment in sputtering chamber. (b) XPS spectra of iridium 4f collected after 2 minutes of surface etching of HSR™ Pt10Ir (top) and oxidized HSR™ Pt10Ir (bottom) electrode. Fitted spectra shown with the red line represents metallic state and fitted spectra shown with the blue line represents Ir^{4+} state of iridium 4f.

Table S1: Binding energy and Area % of iridium metal and Ir^{4+} oxidation state obtained from the iridium 4f spectra of both HSR™ Pt10Ir and oxidized HSR™ Pt10Ir electrodes.

**HSR™-Pt10Ir electrode**		
Spectrum	Binding energy (eV)	Area %
Ir metal 4f 7/2	60.82	58.32
Ir^{4+} 4f 7/2	61.46	26.28
**Oxidized HSR™-Pt10Ir electrode**		
Spectrum	Binding energy (eV)	Area %
Ir metal 4f7/2	60.92	27.25
Ir^{4+} 4f7/2	62.07	56.68
